# Vaccination Status is Not Associated With Adverse Postoperative Outcomes Following Total Joint Arthroplasty in Patients With a Preoperative COVID-19 Diagnosis

**DOI:** 10.1016/j.artd.2025.101673

**Published:** 2025-03-29

**Authors:** Pramod Kamalapathy, Corinne Vennitti, Pradip Ramamurti, James A. Browne

**Affiliations:** University of Virginia, Department of Orthopaedic Surgery, Charlottesville, VA

**Keywords:** COVID vaccination, COVID-19, TKA, THA, Primary total joint arthroplasty

## Abstract

**Background:**

Previous studies have shown that COVID-19 diagnosis increases rates of perioperative infection, readmission, and other complications following surgery. However, the effect of the COVID vaccine in such patients is unknown. We hypothesized that of the patients with COVID diagnosis, vaccinated patients with COVID-19 diagnosis would have lower rates of adverse complications compared to unvaccinated patients undergoing total joint arthroplasty (TJA).

**Methods:**

Using a national database registry, patients aged less than 85 years undergoing elective primary total knee or total hip arthroplasty with at least 90 days of follow-up were included during the first year of COVID-19 pandemic from April 2020-April 2021. Patients were included in the COVID-19 cohort if they had a diagnosis on the day of surgery or within 30 days prior to surgery. Patients with a history of malignancy, joint injection, femoral neck fractures, tibial fractures, and those undergoing revision arthroplasty were excluded from the study. All comparisons were performed using multivariate logistic regression with significance set at *P* < .05. Odds ratio and 95% confidence interval were reported for all comparisons.

**Results:**

There were a total of 1280 patients with COVID-19 diagnosis matched with 3831 patients without COVID-19 diagnosis. Patients with a COVID-19 diagnosis were at an increased risk of pneumonia, acute kidney injury, urinary tract infection, and readmission following TJA compared to patients without COVID-19 diagnosis. However, there were no differences in any complications assessed between vaccinated patients and unvaccinated patients with COVID-19 diagnosis following TJA.

**Conclusions:**

This study confirms that patients with a COVID-19 diagnosis in the 30 days prior to TJA, whether vaccinated or not, have increased risks of medical complications and hospital utilization. However, this study demonstrates that vaccination status does not appear to be associated with the incidence of adverse postoperative events in patients with a COVID-19 diagnosis prior to TJA.

## Introduction

COVID-19 has had a lasting effect on healthcare in the United States; it was estimated that nearly 150 million people tested positive for COVID-19 in the United States by September 2021 [1]. COVID-19 showed that it was a multisystem disease, presenting with a host of clinical manifestations including cardiovascular and respiratory effects. As the COVID-19 pandemic continued to evolve, restrictions lightened, and elective surgery resumed in many areas around the country. While resource utilization and intensive care unit admissions were widely documented, postoperative outcomes remained limited, and it was not initially clear when and how to resume elective surgery [[2], [3], [4]]. Initial reports showed that COVID-19 was associated with an increased risk of infection, kidney injury, cardiopulmonary disease, and increased mortality following surgical procedures [[5], [6], [7], [8]]. Due to the perioperative risk following surgery, many physicians recommended delaying surgery for at least 7 weeks after COVID-19 infection. Some surgeons and researchers suggested that patients should be prioritized for vaccination against COVID before proceeding with elective surgery, even suggesting that vaccination could save lives in this population [7].

Specifically within the arthroplasty literature, an early pandemic study by Forlenza et al showed that COVID-19 positive patients were associated with an increased risk of deep vein thrombosis, pulmonary embolism (PE), renal injury, and urinary tract injury following hip and knee arthroplasty compared to patients without COVID-19 diagnosis [9]. However, the literature on this topic is currently limited by small sample size, and does not account for vaccination status. Many currently published studies were done during the early stages of the pandemic, during which protocols were still being amended around the country, elective surgeries were severely declining, and vaccines were not available. Furthermore, to our knowledge, the question as to whether or not COVID-19 vaccines have decreased postoperative complications in total joint arthroplasty (TJA) remains unaddressed.

Therefore, the objective of the study was to use a national database to assess the (1) impact of COVID-19 diagnosis in patients undergoing total knee arthroplasty (TKA) or total hip arthroplasty (THA) and (2) potential effect of vaccination status on those with positive COVID-19 diagnosis compared to those who did not have COVID-19 diagnosis. This study specifically focuses on the impact of vaccination status within a cohort of patients who had COVID-19 in the perioperative period (within 30 days) prior to TJA. This study does not attempt to analyze the outcomes of those who were vaccinated but did not have COVID-19 prior to TJA, or those who had COVID-19 > 30 days prior to TJA. We hypothesized that patients with COVID diagnosis would have higher rates of infection and medical complications following TJA, while vaccinated patients with COVID-19 diagnosis would have lower rates of adverse complications compared to unvaccinated patients undergoing TJA.

## Material and methods

### Data source

A retrospective database review was performed using commercially available PearlDiver (PearlDiver Inc., Colorado Springs, Colorado, USA; www.pearldiverinc.com) patient records. The PearlDiver Mariner15 dataset contains records of more than 151 million patients across all payer types (commercial, Medicare, Medicaid, government, and cash) from 2010 to 2021, searchable by billable codes. As queried data are deidentified and Health Insurance Portability and Accountability Act compliant, this study was exempt from institutional review board approval. The present study used data of patients undergoing THA or TKA for patient records from April 2020 to April 2021.

### Study population

All patients aged less than 85 years with a COVID-19 diagnosis who underwent elective TJA were identified using Current Procedural Terminology and International Classification of Diseases, 10th Revision codes (Appendix 1). Patients undergoing same-day revision and those with a history of trauma, malignancy, or infection were excluded from the study population. Patients were included in the COVID-19 cohort if they had a COVID-19 diagnosis on the same day of surgery or within 30 days prior to surgery. The control cohort was matched to the COVID-19 group based on age, gender, obesity, tobacco use, opioid use, vaccination history, diabetes, chronic pulmonary disease, hyperlipidemia, hypertension, peripheral vascular disease, chronic kidney disease, coronary artery disease, and congestive heart failure. A subgroup of the COVID-19 cohort was further stratified into those who were vaccinated prior to diagnosis and those who were not vaccinated prior to surgery. Patients who did not have a code suggesting prior vaccination were assumed to be unvaccinated.

### Outcomes

COVID-19 cohort was compared to controls for 90-day medical complications, 30-day and 90-day emergency department visits, and 30-day and 90-day hospital readmissions. One-year infection and revision within 2 years were also queried as well. 90-day medical complications included PE, pneumonia (PNA), myocardial infarction, cerebrovascular accident, sepsis, deep vein thrombosis, acute kidney injury (AKI), urinary tract infection (UTI), transfusion, and wound complications.

### Statistical analysis

Pearson χ^2^ test was used to assess for differences in demographics and pre-existing comorbidities between groups. Comparison of postoperative complications was performed with a multivariate logistic regression after adjusting for demographic factors, including age, sex, obesity, tobacco use history, opioid use history, vaccination status, peripheral vascular disease, diabetes mellitus, congestive heart failure, chronic obstructive pulmonary disease, hyperlipidemia, hypertension, and coronary artery disease. COVID-19 cohort was compared to controls via multivariate regression. Moreover, subgroup analysis was performed comparing vaccinated patients with COVID versus unvaccinated patients with COVID versus patients without COVID. R software embedded within the PearlDiver database (R Foundation for Statistical Computing, Vienna, Austria) was used for all statistical analysis. For all statistical comparisons, *P* < .05 was considered significant.

## Results

The present study identified 1280 patients with a COVID-19 diagnosis prior to surgery and 3381 matched patients without a COVID-19 diagnosis undergoing surgery during the same period. There were no differences between groups in terms of age, gender, comorbidities, or substance use. Both groups had a majority of patients within the age range of 50-64 years. 40.9% of patients were male in the study. The COVID-19 group was comprised of 486 patients (38.0%) undergoing THA and 794 patients (62.0%) undergoing TKA. Similarly, the control group had 1393 patients (36.4%) undergoing THA and 2438 (63.6%) undergoing TKA. There were differences in patients undergoing COVID vaccination prior to surgery (84.4% COVID positive; 48.1% COVID negative) (Table 1).

**Table 1:** Postoperative outcomes of patients with COVID diagnosis compared against those without COVID diagnosis undergoing total joint arthroplasty.

Medical complications (90 d)	COVID diagnosis N = 1280		No COVID diagnosis N = 3831		Statistical analysis		
	n	(%)	n	(%)	OR	95% CI	*P* value
Pulmonary embolism	1	0.1%	1	0.0%	1.44	[0.03-82.3]	.99
Pneumonia	**29**	2.3%	**41**	1.1%	**2.05**	**[1.17-3.64]**	**.012**
Cerebrovascular accident	8	0.6%	21	0.5%	1.06	[0.37-2.74]	.913
Sepsis	15	1.2%	28	0.7%	1.98	[0.92-4.27]	.075
Myocardial infarction	12	0.9%	20	0.5%	1.67	[0.72-3.91]	.228
Deep vein thrombosis	23	1.8%	43	1.1%	1.27	[0.71-2.26]	.418
Acute kidney injury	**34**	2.7%	**70**	1.8%	**1.64**	**[1.00-2.68]**	**.049**
Urinary tract infection	**53**	4.1%	**94**	2.5%	**1.62**	**[1.08-2.42]**	**.018**
Wound complication	14	1.1%	38	1.0%	1.31	[0.62-2.64]	.474
Blood transfusion	8	0.6%	13	0.3%	1.64	[0.58-4.54]	.338
Dysphagia	10	0.8%	18	0.5%	1.89	[0.75-4.64]	.161
Hospital utilization							
Emergency department (30 d)	102	8.0%	253	6.6%	1.05	[0.80-1.37]	.715
Emergency department (90 d)	160	12.5%	360	9.4%	1.21	[0.94-1.52]	.094
Readmission (30 d)	**50**	3.9%	**87**	2.3%	**1.67**	**[1.10-2.53]**	**.008**
Readmission (90 d)	**70**	5.5%	**137**	3.6%	**1.57**	**[1.11-2.23]**	**.009**
Surgical complications							
TJA infection (1 y)	28	2.2%	73	1.9%	1.04	[0.58-1.81]	.894
TJA revision (2 y)	5	0.4%	26	0.7%	0.69	[0.21-1.85]	.485

CI, confidence interval; OR, odds ratio; TJA, total joint arthroplasty.

Bolded lines are findings with a *P* value <.05.

Patients with COVID-19 were associated with an increased risk of PNA (2.3% vs 1.1%; odds ratio [OR]: 2.05; 95% confidence interval [CI]: 1.17-3.64; *P* < .012), AKI (2.7% vs 1.8%; OR: 1.64; 95% CI: 1.00-2.68; *P* = .049), UTI (4.1% vs 2.5%; OR: 1.62; 95% CI: 1.08-2.42; *P* = .018), 30-day readmission (3.9% vs 2.3%; OR: 1.67; 95% CI: 1.10-2.53; *P* = .008), and 90-day readmission (5.5% vs 3.6%; OR: 1.57; 95% CI: 1.11-2.23; *P* = .009) (Table 2). There were no differences in 1-year infection or revision within 2 years following TJA (*P* > .05).

**Table 2:** Comparisons of postoperative outcomes between vaccinated patients with COVID-19 and unvaccinated patients with COVID-19 diagnosis and control group.

Medical complications (90 d)	Vaccinated patient with COVID versus patient without COVID			Vaccinated patient with COVID versus unvaccinated patient with COVID		
	OR	95% CI	*P* value	OR	95% CI	*P* value
Pulmonary embolism	1.44	[0.03-82.3]	.837	-	-	1
Pneumonia	1.78	[0.94-3.41]	.077	0.78	[0.31-2.22]	.609
Cerebrovascular accident	0.81	[0.24-2.39]	.709	0.45	[0.08-3.40]	.37
Sepsis	**2.65**	**[1.12-6.48]**	**.028**	3.18	[0.60-59.5]	.274
Myocardial infarction	1.33	[0.51-3.47]	.556	0.61	[0.98-2.93]	.482
Deep vein thrombosis	1.46	[0.78-2.73]	.237	3.99	[0.82-71.9]	.178
Acute kidney injury	1.47	[0.84-2.57]	.171	0.64	[0.27-1.74]	.352
Urinary tract infection	**1.69**	**[1.09-2.62]**	**.018**	1.72	[0.73-5.05]	.262
Wound complication	1.32	[0.58-2.91]	.489	1.22	[0.30-8.47]	.801
Blood transfusion	1.23	[0.38-3.84]	.718	0.51	[0.11-3.75]	.439
Dysphagia	2.41	[0.77-8.32]	.139	0.66	[0.16-4.50]	.609
Hospital utilization						
Emergency department (30)	0.98	[0.73-1.31]	.882	0.98	[0.57-1.78]	.95
Emergency department (90)	1.11	[0.87-1.42]	.406	0.96	[0.62-1.57]	.891
Readmission	**1.62**	**[1.02-2.56]**	**.037**	1.23	[0.57-3.08]	.615
Readmission (90)	**1.53**	**[1.05-2.24]**	**.028**	1.18	[0.61-2.53]	.638
Surgical complications						
TJA infection (1 y)	0.9	[0.54-1.99]	.726	0.75	[0.31-2.11]	.561
TJA revision (2 y)	0.5	[0.11-1.70]	.301	0.24	[0.04-2.07]	.152

CI, confidence interval; OR, odds ratio; TJA, total joint arthroplasty.

Bolded lines are findings with a *P* value <.05.

### Vaccination status

The COVID-19 group was subdivided into those who were vaccinated prior to diagnosis (N = 1080) and those who were not vaccinated (N = 200). There were no differences in age, gender, or comorbidities between those who were vaccinated with COVID-19 diagnosis, those who were unvaccinated with COVID-19 diagnosis, and those who did not have COVID-19 diagnosis. Multivariate regression showed that vaccinated patients with COVID-19 had an increased risk of sepsis (OR: 2.65; 95% CI: 1.12-6.48), UTI (OR: 1.69; 95% CI: 1.09-2.62; *P* = .018), 30-day readmission (OR: 1.62; 95% CI: 1.02-2.56), and 90-day readmission (OR: 1.53; 95% CI: 1.05-2.24) when compared to patients without COVID-19. Multivariate regression showed that there were no differences in medical or surgical outcomes between those with COVID-19 regardless of vaccination status (*P* > .05).

## Discussion

Although COVID-19 has moved from pandemic to endemic, questions remain regarding how to safely proceed with elective TJA surgery. An early pandemic study showed that COVID-19 diagnosis was associated with an increased risk of adverse complications, such as UTI, renal injury, and infection following TJA [10]. Our study confirmed these findings, suggesting that a preoperative COVID-19 diagnosis in the month before surgery is associated with an increased risk of PNA (OR: 2.05; 95% CI: 1.17-3.64), AKI (OR: 1.64; 95% CI: 1.00-2.68), and UTI (OR: 1.62; 95% CI: 1.08-2.42) following TJA. The novel finding of this study was that vaccination status was not associated with differences in postoperative outcomes or complications in patients with a preoperative COVID-19 diagnosis. This runs contrary to what has been suggested by others [7].

Previously published literature has examined the impact of recent COVID-19 infection on postoperative outcomes after various elective surgeries. In a study of 5479 patients from the COVID-19 research database undergoing a variety of major elective surgical procedures, patients classified as “peri-COVID-19” (defined as surgery within 0-4 weeks after initial diagnosis) had increased odds of developing postoperative PNA (OR: 6.46; 95% CI: 4.06-6.16), respiratory failure (OR: 3.36; 95% CI: 2.22-5.10), PE (OR: 2.73; 95% CI: 1.35-5.53), and sepsis (OR: 3.67; 95% CI: 2.18-6.16) compared to matched patients undergoing the same procedures prior to January 2020 [11]. Similar results showing increased rates of pulmonary complications and mortality, among other complications, for patients with recent COVID-19 infection have been reported, as well [[5], [6], [7], [8]].

The association between COVID-19 and risk of coagulopathy is under investigation with retrospective reviews demonstrating an increased risk of venous thromboembolism following surgery [[12], [13], [14]]. This is particularly relevant to patients undergoing TJA, who are already at an increased risk compared to other surgical procedures [15]. While the reason for the absence of this relationship in the present study is not entirely clear, it is possible that timing and severity of infection play a role in venous thromboembolism risk, with more severe or recent infections carrying higher risk [10,16]. The present study was not able to differentiate the severity of illness as well as differentiate symptomatic versus asymptomatic diagnosis as patients could have been tested as part of routine screening prior to surgery.

The present study also investigated whether COVID-19 vaccination, among patients with a diagnosis of COVID-19, would have any impact on rates of postoperative complications. There were no differences in postoperative outcomes in patients with COVID regardless of vaccination status. Vaccinated patients with COVID were also at an increased risk of sepsis and readmission following surgery compared to those without COVID-19. These results conflict with previously published studies that show some protective effect of full or even partial COVID-19 vaccination on postoperative pulmonary and thrombotic complications, as well as positive effects on length of stay [17,18]. These studies were not looking specifically at arthroplasty procedures. It is also possible that vaccination, while protective against new COVID-19 infection following surgery, does not confer additional benefit to patients who develop active infection prior to surgery. It is also worth noting that, due to the nature of the dataset used for this study, it was not possible to determine precise vaccination status for all patients, or which formulation was given, both of which could impact the results [19,20].

From a hospital utilization standpoint, this study demonstrates that a COVID-19 diagnosis was associated with significantly increased odds of readmission at both 30 and 90 days following surgery regardless of vaccination status. This trend has been observed in the literature for elective orthopaedic surgery as well as other surgical specialties during the peak of the COVID-19 pandemic [[21], [22], [23]]. Certainly the COVID infection itself may lead to medical complications that result in readmission. Other potential causes behind this association could be, among other factors, suboptimal care from strained resources or staffing for COVID-19 positive patients, efforts to discharge patients quickly to minimize potential COVID-19 exposure, or reluctance to discharge patients to outpatient rehabilitation facilities due to COVID-19 exposure risk. Regardless of the underlying reason for readmission, increased readmission rates present a burden for both the healthcare system and patients.

The present study has several limitations that warrant discussion. The data were retrieved from a large database using International Classification of Diseases and Current Procedural Terminology codes; therefore, human errors in code imputation are inherent. The accuracy of coding for both COVID-19 infection as well as vaccination is unknown. Additionally, timing and type of vaccination was unable to be determined using the data retrieved. Patients with active COVID-19 infection may lack a diagnosis due to inadequate or absent testing. The study was unable to differentiate between symptomatic and asymptomatic COVID-19 diagnosis. It is also possible that some vaccinated patients did not have an administrative code registered in their chart and thus would have been misclassified as unvaccinated in this study. Finally, the nature of both the vaccines and COVID-19 virus change over time, so the conclusions of the study are limited to the particular time period studied.

Despite these limitations, this study offers valuable insight into the impact of recent COVID-19 infection on postoperative outcomes for TJA in a large, nationally representative sample. Future research must assess surgical timing following COVID-19 diagnosis. While this is already generally accepted practice, there is still no consensus regarding optimal timing. A prior study published by Baiocchi et al. found that patients with preoperative asymptomatic COVID-19 infection did not have increased rates of complications when surgery was delayed by 25 days, compared to patients without a positive COVID-19 test [24]. A second study by COVID-19 Surg Collaborative found that rates of pulmonary complications were lowest when surgery was delayed by at least 7 weeks following a positive COVID-19 test [10]. While the present study only included patients with a diagnosis of COVID-19 within 30 days of surgery, the previously mentioned studies support the current results that complication rates are increased for at least 4 weeks after initial diagnosis. Important differences to consider between these studies and the present study are that both Baiocchi et al. and the COVID-19 Surg Collaborative included a variety of operations in their studies, and the COVID-19 Surg Collaborative also included nonelective procedures. Because different procedures involve unique risk profiles, the current results are specific to the elective total joint population.

Further work is also necessary to completely understand the association between vaccination status and outcomes in patients with and without a COVID diagnosis, particularly as the severity of COVID infection changes as the pandemic transitions into an endemic state. Additional comparison groups can also be analyzed including comparisons between all combinations of vaccination status and diagnosis of COVID-19 to gather further data regarding combinations with increased postoperative complications. As we have an increasing cohort of patients who were diagnosed with COVID-19 more than 1 year prior to surgery, analysis on the impact of COVID-19 within 90 days and 1 year prior to TJA can be obtained. Finally, no analysis of the strain of COVID-19 is available currently, so subtype analysis cannot be completed. Future studies including larger sample sizes, longer timeframe, and further strain analysis should be considered to provide more data for additional cross comparisons.

## Conclusions

The present study shows that patients with a COVID-19 diagnosis in the 30 days prior to TJA in the time period addressed with this cohort, whether vaccinated or not, had increased risk of medical complications and hospital utilization compared to patients without a COVID-19 diagnosis. This, however, cannot necessarily be generalized to current strains due to the evolving nature of the virus and inability to identify and analyze different strains of COVID-19 within our cohort. There were no differences in adverse events between vaccinated and unvaccinated patients in the COVID-19 group following TJA. These findings do not support the use of vaccination status as a risk factor for TJA in patients with a recent COVID diagnosis.

## CRediT authorship contribution statement

**Pramod Kamalapathy:** Writing – original draft, Methodology, Formal analysis, Data curation. **Corinne Vennitti:** Writing – review & editing, Writing – original draft. **Pradip Ramamurti:** Writing – original draft, Investigation, Formal analysis, Data curation. **James A. Browne:** Writing – review & editing, Validation, Supervision, Conceptualization.

## Conflicts of interest

James A. Browne received royalties from Enovis; is a paid consultant for Enovis, Ortho-DX, Kinamed, and Orthoremedies; holds stock or stock options in Radlink and Ortho-DX; received royalties, financial or material support from the Journal of Arthroplasty, Journal of Bone and Joint Surgery, and Saunders/Mosby-Elsevier; is in the medical/orthopaedic publications editorial/governing board of Journal of Arthroplasty; and is a board member of AAHKS, AJRR, Hip Society, Knee Society, and SOA. All other authors declare no potential conflicts of interest.

For full disclosure statements refer to https://doi.org/10.1016/j.artd.2025.101673.

## Appendix A Supplementary data

Conflict of Interest Statement for Browne

Conflict of Interest Statement for Ramamurti

Conflict of Interest Statement for Kamalapathy

Conflict of Interest Statement for Vennitti
