# Supplementary material for: Vaccination Status is Not Associated With Adverse Postoperative Outcomes Following Total Joint Arthroplasty in Patients With a Preoperative COVID-19 Diagnosis
Source: Arthroplast Today. 2025 Mar 29;33:101673. doi: 10.1016/j.artd.2025.101673 (PMC11995801; doi:10.1016/j.artd.2025.101673)
Supplement: Conflict of Interest Statement for Browne [file mmc1.pdf]

CONFLICT OF INTEREST STATEMENT

American Association of Hip and Knee Surgeons

(Adopted from the American Academy of Orthopaedic Surgeons disclosure statement)

The following form **must be filled out completely and submitted by each author (example, 6 authors, 6 forms). All items require a response. If there is no relevant disclosure for a given item, enter "*None*."**

Vaccination Status is not Associated with Adverse Postoperative Outcomes Following Total Joint Arthroplasty in Patients with a Preoperative COVID-19 Diagnosis

Manuscript Title

1. Royalties from a company or supplier (The following conflicts were disclosed)

   Yes: Enovis

2. Speakers bureau/paid presentations for a company or supplier (The following conflicts were disclosed)

   none

3A. Paid employee for a company or supplier (The following conflicts were disclosed)

   none

3B. Paid consultant for a company or supplier (The following conflicts were disclosed)

   Yes: Enovis, Ortho-DX, Kinamed, Orthoremedies

3C. Unpaid consultants for a company or supplier (The following conflicts were disclosed)

   none

4. Stock or stock options in a company or supplier (The following conflicts were disclosed)

   Yes: Radlink, Ortho-DX

5. Research support from a company or supplier as a Principal Investigator (The following conflicts were disclosed)

   none

6. Other financial or material support from a company or supplier (The following conflicts were disclosed)

   none

7. Royalties, financial or material support from publishers (The following conflicts were disclosed)

   Yes: Journal of Arthroplasty, Journal of Bone and Joint Surgery, Saunders/Mosby-Elsevier

8. Medical/Orthopaedic publications editorial/governing board (The following conflicts were disclosed)

   Yes: Journal of Arthroplasty

9. Board member/committee appointments for a society (The following conflicts were disclosed)
   Yes: AAHKS, AJRR, Hip Society, Knee Society, SOA

Each author must sign AND print or type his/her name, date and submit a separate form

In addition, one BLINDED Conflict of Interest form (no author names used) should be submitted per manuscript with all author disclosures.

James A. Browne	James A. Browne	10/6/2024
Author Name (Print or Type)	Author Signature	Date
