# Supplementary material for: Vaccination Status is Not Associated With Adverse Postoperative Outcomes Following Total Joint Arthroplasty in Patients With a Preoperative COVID-19 Diagnosis
Source: Arthroplast Today. 2025 Mar 29;33:101673. doi: 10.1016/j.artd.2025.101673 (PMC11995801; doi:10.1016/j.artd.2025.101673)
Supplement: Supplemental Table 1 [file mmc5.docx]

|  | ***COVID diagnosis*** | | ***No COVID diagnosis*** | | ***P - value*** |
| --- | --- | --- | --- | --- | --- |
|  | ***N = 1,280*** | | ***N = 3,831*** | |  |
| **Demographics** | **n** | **(%)** | **n** | **(%)** |  |
| Age Group |  | | | | |
| *Under 50* | 84 | 6.56% | 252 | 6.58% | 1.000 |
| *50-64 years* | 533 | 41.64% | 1595 | 41.63% |  |
| *65-74 years* | 443 | 34.61% | 1328 | 34.66% |  |
| *74-85 years* | 220 | 17.19% | 656 | 17.12% |  |
| Sex (male) | 523 | 40.86% | 1,565 | 40.85% | 1.000 |
| **Comorbidities** | **n** | **(%)** | **n** | **(%)** | ***P - value*** |
| Obesity (BMI >30 kg/m^2^) | 545 | 42.58% | 1,630 | 42.55% | 1.000 |
| Diabetes Mellitus | 240 | 18.75% | 717 | 18.72% | 1.000 |
| Hyperlipidemia | 385 | 30.08% | 1,149 | 29.99% | 0.982 |
| Hypertension | 985 | 76.95% | 2,949 | 76.98% | 1.000 |
| Peripheral Vascular disease | 170 | 13.28% | 490 | 12.79% | 0.685 |
| Congestive Heart Failure | 54 | 4.22% | 156 | 4.07% | 0.883 |
| Coronary Artery Disease | 310 | 24.22% | 925 | 24.15% | 0.987 |
| Chronic Kidney Disease | 176 | 13.75% | 523 | 13.65% | 0.967 |
| Chronic Lung Disease | 346 | 27.03% | 1,033 | 26.96% | 0.992 |
| Depression | 411 | 32.11% | 1,219 | 31.82% | 0.874 |
| **Substance Use** | **n** | **(%)** | **n** | **(%)** | ***P - value*** |
| Tobacco Use | 419 | 32.73% | 1,250 | 32.63% | 0.972 |
| Opioids | 387 | 30.23% | 1,180 | 30.80% | 0.723 |
| **Surgical Factors** | **n** | **(%)** | **n** | **(%)** | ***P - value*** |
| Total Hip Arthroplasty | 486 | 37.97% | 1,393 | 36.36% | 0.301 |
| Total Knee Arthroplasty | 794 | 62.03% | 2,438 | 63.64% |  |
| COVID vaccine | 1,080 | 84.38% | 1,844 | 48.13% | <0.001 |

SDC1 Patient Demographics of Patients with COVID Diagnosis and those without COVID Diagnosis Undergoing Elective TJA
