# Supplementary material for: Vaccination Status is Not Associated With Adverse Postoperative Outcomes Following Total Joint Arthroplasty in Patients With a Preoperative COVID-19 Diagnosis
Source: Arthroplast Today. 2025 Mar 29;33:101673. doi: 10.1016/j.artd.2025.101673 (PMC11995801; doi:10.1016/j.artd.2025.101673)
Supplement: Supplemental Table 2 [file mmc6.docx]

|  | ***Vaccinated patient with COVID Diagnosis*** | | ***Unvaccinated patient with COVID diagnosis*** | | ***No COVID diagnosis*** | | ***P-value*** |
| --- | --- | --- | --- | --- | --- | --- | --- |
|  | ***N = 1,080*** | | ***N = 200*** | | ***N = 3831*** | |  |
| **Demographics** | **n** | **(%)** | **n** | **(%)** | **n** | **(%)** |  |
| Age Group |  | | | | | | |
| *Under 50* | 78 | 7.22% | 11 | 5.50% | 252 | 6.58% | 0.996 |
| *50-64 years* | 450 | 41.67% | 83 | 41.50% | 1595 | 41.63% |  |
| *65-74 years* | 373 | 34.54% | 70 | 35.00% | 1328 | 34.66% |  |
| *74-85 years* | 179 | 16.57% | 36 | 18.00% | 656 | 17.12% |  |
| Sex (male) | 434 | 40.19% | 89 | 44.50% | 1,565 | 40.85% | 0.522 |
| **Comorbidities** | **n** | **(%)** | **n** | **(%)** | **n** | **(%)** | ***P-value*** |
| Obesity (BMI >30 kg/m^2^) | 469 | 43.43% | 76 | 38.00% | 1,630 | 42.55% | 0.362 |
| Diabetes Mellitus | 200 | 18.52% | 40 | 20.00% | 717 | 18.72% | 0.885 |
| Hyperlipidemia | 336 | 31.11% | 49 | 24.50% | 1,149 | 29.99% | 0.173 |
| Hypertension | 834 | 77.22% | 151 | 75.50% | 2,949 | 76.98% | 0.868 |
| Peripheral Vascular disease | 144 | 13.33% | 26 | 13.00% | 518 | 13.52% | 0.968 |
| Congestive Heart Failure | 42 | 3.89% | 12 | 6.00% | 156 | 4.07% | 0.375 |
| Coronary Artery Disease | 257 | 23.80% | 53 | 26.50% | 925 | 24.15% | 0.713 |
| Chronic Kidney Disease | 153 | 14.17% | 23 | 11.50% | 523 | 13.65% | 0.599 |
| Chronic Lung Disease | 288 | 26.67% | 58 | 29.00% | 1,033 | 26.96% | 0.792 |
| Depression | 358 | 33.15% | 53 | 26.50% | 1,305 | 34.06% | 0.083 |
| **Substance Use** | **n** | **(%)** | **n** | **(%)** | **n** | **(%)** | ***P-value*** |
| Tobacco Use | 363 | 33.61% | 56 | 28.00% | 1,250 | 32.63% | 0.298 |
| Opioids | 328 | 30.37% | 59 | 29.50% | 1,194 | 31.17% | 0.798 |
| **Surgical Factors** | **n** | **(%)** | **n** | **(%)** | **n** | **(%)** | ***P-value*** |
| Total Hip Arthroplasty | 410 | 37.96% | 76 | 38.00% | 1,393 | 36.36% | 0.587 |
| Total Knee Arthroplasty | 670 | 62.04% | 124 | 62.00% | 2,438 | 63.64% |  |
| COVID vaccine | 1,080 | 100.00% | 0 | 0.00% | 1,844 | 48.13% | <0.001 |

Supplemental Table 2. Patient Demographics of Vaccinated Patients with COVID-19 diagnosis, Unvaccinated Patients with COVID-19 Diagnosis, and Controls undergoing Total Joint Arthroplasty
