# Supplementary material for: Vaccination Status is Not Associated With Adverse Postoperative Outcomes Following Total Joint Arthroplasty in Patients With a Preoperative COVID-19 Diagnosis
Source: Arthroplast Today. 2025 Mar 29;33:101673. doi: 10.1016/j.artd.2025.101673 (PMC11995801; doi:10.1016/j.artd.2025.101673)
Supplement: Supplemental Table 3 [file mmc7.docx]

|  | ***Vaccinated patient with COVID Diagnosis***  ***N = 1,080*** | | ***Unvaccinated patient with COVID diagnosis***  ***N = 200*** | |
| --- | --- | --- | --- | --- |
| ***Medical Complications (90 days)*** | **n** | **(%)** | **n** | **(%)** |
| Pulmonary Embolism | 1 | 0.09% | 0 | 0.00% |
| Pneumonia | 23 | 2.13% | 6 | 3.00% |
| Cerebrovascular Accident | 5 | 0.46% | 2 | 1.00% |
| Sepsis | 14 | 1.30% | 1 | 0.50% |
| Myocardial Infarction | 9 | 0.83% | 3 | 1.50% |
| Deep Vein Thrombosis | 22 | 2.04% | 1 | 0.50% |
| Acute Kidney Injury | 27 | 2.50% | 7 | 3.50% |
| Urinary Tract Infection | 48 | 4.44% | 5 | 2.50% |
| Wound Complication | 12 | 1.11% | 2 | 1.00% |
| Blood Transfusion | 6 | 0.56% | 2 | 1.00% |
| Dysphagia | 8 | 0.74% | 2 | 1.00% |
| ***Hospital Utilization*** | **n** | **(%)** | **n** | **(%)** |
| Emergency Department (30 days) | 86 | 7.96% | 16 | 8.00% |
| Emergency Department (90 days) | 135 | 12.50% | 25 | 12.50% |
| Readmission (30 days) | 43 | 3.98% | 7 | 3.50% |
| Readmission (90 days) | 60 | 5.56% | 10 | 5.00% |
| ***Surgical Complications*** | **n** | **(%)** | **n** | **(%)** |
| TJA Infection (1 Year) | 22 | 2.04% | 6 | 3.00% |
| TJA Revision (2 Year) | 3 | 0.28% | 2 | 1.00% |

Supplemental Table 3. Postoperative Outcomes of Vaccinated and Unvaccinated Patients with COVID-19 Diagnosis undergoing Total Joint Arthroplasty
